# A comprehensive biomechanical evaluation of length and diameter of dental implants using finite element analyses: A systematic review

**DOI:** 10.1016/j.heliyon.2024.e26876

**Published:** 2024-02-22

**Authors:** Piaopiao Qiu, Rongkai Cao, Zhaoyang Li, Zhen Fan

**Affiliations:** a Department of Implantology, Stomatological Hospital and Dental School of Tongji University, Shanghai Engineering Research Center of Tooth Restoration and Regeneration, Shanghai, China; b Stomatological Hospital and Dental School of Tongji University, Shanghai Engineering Research Center of Tooth Restoration and Regeneration, Shanghai, China

**Keywords:** Biomechanical evaluation, **Length**, **Diameter**, **Finite element analyses**, **Dental implant**

## Abstract

**Background:**

With a wide range of dental implants currently used in clinical scenarios, evidence is limited on selecting the type of dental implant best suited to endure the biting force of missing teeth. Finite Element Analysis (FEA) is a reliable technology which has been applied in dental implantology to study the distribution of biomechanical stress within the bone and dental implants.

**Purpose:**

This study aimed to perform a systematic review to evaluate the biomechanical properties of dental implants regarding their length and diameter using FEA.

**Material and methods:**

A comprehensive search was performed in PubMed/MEDLINE, Scopus, Embase, and Web of Science for peer-reviewed studies published in English from October 2003 to October 2023. Data were organized based on the following topics: area, bone layers, type of bone, design of implant, implant material, diameter of implant, length of implant, stress units, type of loading, experimental validation, convergence analysis, boundary conditions, parts of Finite Element Model, stability factor, study variables, and main findings. The present study is registered in PROSPERO under number CRD42022382211.

**Results:**

The query yielded 852 results, of which 40 studies met the inclusion criteria and were selected in this study. The diameter and length of the dental implants were found to significantly influence the stress distribution in cortical and cancellous bone, respectively. Implant diameter was identified as a key factor in minimizing peri-implant stress concentrations and avoiding crestal overloading. In terms of stress reduction, implant length becomes increasingly important as bone density decreases.

**Conclusions:**

The diameter of dental implants is more important than implant length in reducing bone stress distribution and improving implant stability under both static and immediate loading conditions. Short implants with a larger diameter were found to generate lower stresses than longer implants with a smaller diameter. Other potential influential design factors including implant system, cantilever length, thread features, and abutment collar height should also be considered in future implant design as they may also have an impact on implant performance.

## 1 Introduction

Over the past decades, dental implants have become a reliable option for the treatment of missing teeth and the improvement of life quality, as evidenced by several clinical studies [[1], [2], [3]]. However, in cases where there is alveolar crest atrophy with insufficient bone height and width, regular dental implants cannot be placed without additional bone augmentation [4,5]. Multiple research efforts have been proposed to simplify the procedure and minimize complications under these situations [6]. Consequently, narrow or short implants, which are usually defined as implants with a diameter between 3 and 3.5 mm or shorter than 10 mm, have been recommended as a solution for these challenging clinical situations, and their popularity in dental implantology is increasing [7,8]. In many cases, the use of narrow or short implants can significantly reduce patient morbidity and allow for quicker definitive prosthetic rehabilitation.

Despite the advantages of narrow or short implants, their application is limited. These implants have smaller contact areas with the bone compared to standard implants, which may result in biomechanical instability and reduced mechanical strength, particularly in high occlusal load areas [9]. Various clinical and experimental studies have examined the shortcomings by evaluating the key factors of implant success [10]. Implant parameters including implant diameter and length are in the spotlight [11]. Due to the decreased length of short implants and the reduced diameter of narrow implants, their clinical use in fixed restorations must be carefully reviewed. In addition, despite the success of implantation, marginal bone loss (MBL) may occur, which remains a major complication and a controversial issue in bone and oral health [12].

Although traditional methods, such as strain gauge and photoelastic stress analysis, have considerably advanced the evaluation of stress distribution, they display limitations. For example, strain gauges could only record the strains on a specific surface, which may have some limitations due to the geometry of the structure they bonded [13]. Similarly, the results of photoelastic stress analysis are limited in the dental community due to their characteristics. FEA is a reliable approach for biomechanical evaluation in dental implant research to determine the distribution of stress affecting dental implants due to its multiple advantages over traditional methods. FEA to produce quantitative and qualitative biomechanical data in dentistry has received multiple attentions as they are effective in assessing stresses and load distribution on the restorations, implants, and peri-implant tissues under functional forces. It allows for the exploration of certain parameters, such as implant length and diameter, through iterative analysis with no ethical implications that would be difficult to achieve in clinical settings [14]. Other advantages of FEA in dentistry encompass their ability to be applied for high-throughput analysis and the mimicking of complex structures showing irregular geometry [15]. For example, the stress distribution at the bone and implant level in the case of MBL could also be studied with the help of FEA [12]. With the help of FEA, clinicians may evaluate stress distribution in the contact area between the surrounding bone and dental implants, which could be a critical part of the success of implantation [16].

However, since FEA is an in-silico numerical analysis, certain limitations must be considered when evaluating its results before a clinical decision. The absence of pH simulation, temperature, biofilm, and the use of isotropic materials are examples of limitations that should be taken into account when evaluating FEA results [17]. In addition, FEA requires detailed modeling and multifaceted scheming with correct boundary conditions [18]. In addition to FEA, some other computational methods like machine learning and deep learning have also been applied in some recent research. For example, one study evaluated two automatic systems classifying the size of implants based on periapical radiographs with deep learning and clustering [19]. Another study developed a machine learning model that can predict the failure of dental implants and peri-implantitis as a tool for maximizing the success of dental implants [20].

The appropriate choice of implant diameter and length would reduce stress distribution in cancellous bone, leading to a reduction in further bone resorption [21]. Although extensive studies have been performed in this area, a conclusive conclusion has not been drawn, especially with a comprehensive consideration of both the length and diameter of dental implants. Accordingly, it is therefore important to elucidate the specific roles of the length and diameter of dental implants and the extent of their effects. The objective of this study was to sum up the current literature and to give a comprehensive consideration of both the diameter and length of dental implants concerning biomechanical properties using finite element analyses. The hypothesis of this study was that the implant diameter is more important than the implant length in the stress distribution of dental implants.

## 2 Materials and methods

This study was registered at PROSPERO under number CRD42022382211 and performed according to the Preferred Reporting Items for Systematic Reviews and Meta-analyses (PRISMA) guidelines [22].

The guiding question was formulated through the PICO format, with (P) presenting the participants, (I) indicating the intervention, (C) standing for the comparison, and (O) representing the outcome [23]. Specifically, it was, “In scenarios with partially edentulous (P), what are the influences of dental implants (I) that are designed with various diameters and lengths (C) on the stress distribution during function evaluated by Finite Element Models (O)?”

### 2.1 Search strategy

An extensive search was performed in PubMed/MEDLINE, Embase, Web of Science, and Scopus for studies published from October 1993 to October 2023. The keywords used were: (finite element analysis) AND (dental implant) AND (diameter) AND (length). The specific search strategies used for each database have been provided in Table 1. Gray literature searches were also performed on the SciELO and Open Access Theses and Dissertations. To complement the study, a manual search of the reference lists from selected articles was supplemented with the database search.

**Table 1:** Electronic databases used and search strategies.

Database	Search strategy
PubMed	("finite element analysis" [MeSH Terms] OR ("finite" [All Fields] AND "element" [All Fields] AND "analysis" [All Fields]) OR "finite element analysis" [All Fields]) AND ("dental implants" [MeSH Terms] OR ("dental" [All Fields] AND "implants" [All Fields]) OR "dental implants" [All Fields] OR ("dental" [All Fields] AND "implant" [All Fields]) OR "dental implant" [All Fields]) AND ("diameter" [All Fields] OR "diameters" [All Fields]) AND ("length" [All Fields] OR "lengths" [All Fields])
Embase	('finite element analysis'/exp OR 'finite element analysis' OR (finite AND ('element'/exp OR element) AND ('analysis'/exp OR analysis))) AND ('dental implant'/exp OR 'dental implant' OR (('dental'/exp OR dental) AND ('implant'/exp OR implant))) AND ('diameter'/exp OR diameter) AND ('length'/exp OR length)
Scopus	(TITLE-ABS-KEY (finite AND element AND analysis) AND TITLE-ABS-KEY (dental AND implant) AND TITLE-ABS-KEY (diameter) AND TITLE-ABS-KEY (length))
Web of Science	(TS=(finite element analysis) AND TS=(dental implant) AND (TS=(diameter) OR TS=(diameters)) AND (TS=(length) OR TS=(lengths)))

### 2.2 Eligibility criteria

The following inclusion criteria were performed to identify publications: (1) peer-reviewed research publications written in English; (2) in vitro mathematical studies; and (3) studies that evaluated the biomechanical properties of both implant length and diameter using finite element analysis.

Studies were excluded based on the following criteria: (1) literature reviews, prospective studies, in-vivo studies, retrospective studies, and animal studies; (2) studies written in other languages; (3) research on orthodontic implants; and (4) studies that did not include dental implants with a diameter between 3 and 3.5 mm or shorter than 10 mm.

### 2.3 Study selection

After unifying relevant information and removing duplicate entries, the abstract and title of returned publications were assessed according to eligibility criteria by two reviewers independently. Articles assessed ineligible by both reviewers were immediately excluded, while articles considered ineligible by one reviewer but eligible by another were retained for full text reading. Two investigators working together read all full-text articles not excluded. Those eligible articles were retained to perform data extraction. Any disagreements were further resolved by discussion with all authors to reach an agreement.

Data from the included studies were gathered meticulously. A report of the following information was extracted: author(s), year of publication, area, bone layers, type of bone, design of implant, implant material, diameter of implant, length of implant, stress units, type of loading, experimental validation, convergence analysis, boundary conditions, parts of Finite Element Model, stability factor, study variables, and main findings.

### 2.4 Quality assessment

The quality assessment was performed in accordance with a previous research [24]. The included studies were scored in accordance with six predefined criteria, including design of the implant model, design of the prosthetic restoration, bone model, type of loading, number of elements, and model dimensions (Table 2).

**Table 2:** Criteria for quality assessment.

Criteria	1 pt	2 pts	3 pts
Implant model	Poor	Complex	Very complex
Bone model	Poor	Complex	Very complex
Element number	<50,000	50,000–100,000	>100,000
Type of loading	Axial	Multiple directions	–
Prosthetic restoration	Crown/bridge	–	–
Dimensions	3D	–	–

## 3 Results

### 3.1 Study selection

**Fig. 1:**
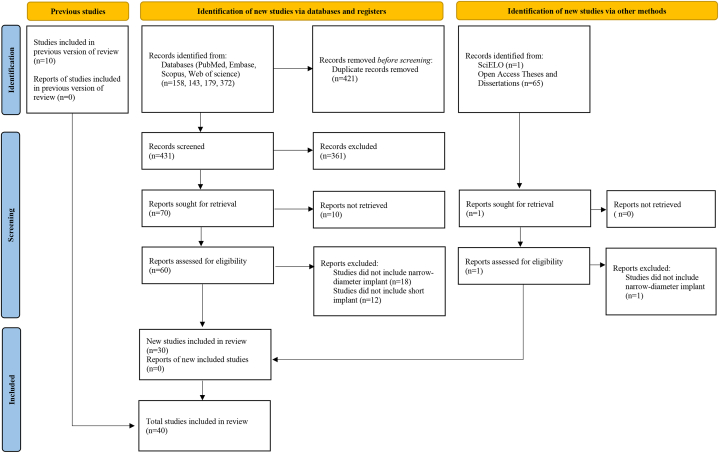
Flow chart of the literature research and results.

A total of 852 references were retrieved from the database (158 from PubMed/MEDLINE, 143 from Embase, 179 from Scopus, and 372 from Web of Science. After removing duplicates, 431 studies were left, and 361 of these were excluded after evaluating their titles and abstracts. 30 studies were further excluded upon full-text reading for not meeting the eligibility criteria. None of the 66 studies obtained from the gray literature was considered eligible. Eventually, 40 studies were included in this systematic review (Fig. 1).

### 3.2 Characteristics of the studies

A summary of the data extracted from the selected researches is displayed in Table 3. In terms of the area of dental implants, both maxilla and mandible models were evaluated in selected studies, and most studies evaluated the stress distribution of dental implants in the posterior region. Regarding the bone models, all applied cortical-layered and trabecular-layered bone models. Implant diameter and length also varied in selected researches due to their different focus and objectives. Type II bone was the most widely used bone type in the literature, and other kinds of bone were also programmed. The implant design varied among the studies such as threaded, cylindrical, screwed, or tapered implants, with most using pure titanium implants. For the measurement of the stress distribution, von Mises MPa was applied in dominant studies, while other studies with tangential stress were recorded in MPa or strain in μStrain. As for the loading conditions, static loading was applied in most studies since only 5 studies utilized immediate loading in the FEA models. Axial load was applied in 17 studies, while the other 23 studies reported loading in multiple directions. Among the 40 studies included, only one study conducted experimental validation for FEM and 11 researches used convergence analysis. Boundary condition was applied in most included studies by constraining the displacement of the nodes in all directions.

**Table 3:** Summary characteristics of the included studies.

Author (year)	Area	Bone layers	Type of bone	Design of implant	Implant material	Diameter (mm)	Length (mm)	Stress units
Alqahtani et al. [25] 2023	Posterior maxilla	C + T	D4	Threaded	Titanium	4, 5, 6	6	von Mises, strains
Anitua et al. [26] 2010	ND	ND	ND	Cylindrical	Titanium	2.5, 3.3, 3.75, 4.0, 4.5, 5.0	8.5, 10, 11.5, 13,15	Mpa
Baggi et al. [27] 2008	Maxilla and mandible	C + T	II	Threaded	Ti–6Al–4V	3.3 to 4.5	11 to 17	Mpa
Balkaya et al. [28] 2014	Mandible	C + T	ND	ND	Titanium	3.5, 5.5	8, 10, 12, 14, 16	von Mises MPa
Bayrak et al. [29] 2020	Mandible	C + T	ND	Cylindrical	Titanium	3.5, 4.5	6, 10.5	von Mises MPa
Borie et al. [30] 2016	Anterior maxilla	C + T	II	Conical	Ti–6Al–4V	3.75, 4	8.5, 10	Mpa
Bourauel et al. [31] 2012	Mandible	C + T	ND	Threaded	Titanium	1.8, 2.1, 2.4,2.5, 2.9, 3.0, 3.3, 3.5, 4, 4.1, 5, 6	5, 5.7, 6, 8, 9, 10,13,15,16,18.5,	von Mises MPa
Chakraborty et al. [32] 2022	Mandible	C + T	I, II, III, IV	Cylindrical, Threaded	Titanium	2.7, 3.5, 4.1	10, 13, 15	μStrain
Demenko et al. [33] 2014	Posterior mandible	C + T	ND	Cylinder-Line	Ti–6Al–4V	2.5 to 7	3 to 17	von Mises MPa
Demenko et al. [34] 2019	Posterior maxilla	C + T	IV	ND	Titanium	3.3, 4.1, 4.8, 5.4	4.5, 5.5, 6.5, 7.5, 8.5,14.5	von Mises MPa
Ding et al. [35] 2009	Mandible	C + T	ND	Threaded	Titanium	3.3 to 4.8	6 to 14	Mpa
Ding et al. [36] 2009	Mandible	C + T	ND	Screwed	Titanium	3.3,4.1,4.8	10	von Mises MPa
Eazhil et al. [37] 2016	Posterior mandible	C + T	ND	Threaded	Ti–6Al–4V	3.5, 4.3, 5	10, 13, 16	von Mises MPa
Elleuch et al. [38] 2021	Mandible	C + T	ND	Threaded	Ti6Al4V	3 to 6.5	10 to 18	von Mises MPa
Faegh et al. [39] 2010	Anterior mandible	C + T	ND	Threaded, no Thread	ND	3	ND	von Mises MPa
Forna et al. [40] 2020	Mandible	C + T	II	Tapered, threaded	Ti–6Al–4V	3.3 to 6.0	5 to 13	von Mises MPa
Georgiopoulos et al. [41] 2007	Mandibular premolar	C + T	ND	Cylindrical	Titanium	3, 3.75, 4.5, 5	8, 10, 12, 14	von Mises MPa
Guan et al. [42] 2010	Mandible	C + T	ND	Threaded	ND	3.5, 4.0, 4.5, 5.5	7, 9, 11, 13, 15	von Mises MPa
Gümrükçü et al. [43] 2018	Maxilla	C + T	III	Cylinder screwed	Chromium-nickel	4.1	6, 8, 11.5, 13, 16	von Mises MPa
Güzelce et al. [44] 2023	Mandibular premolar	C + T	II	ND	Ti–6Al–4V	2.4, 4.1	12	von Mises MPa
Himmlová et al. [45] 2004	Posterior mandible	C	ND	Cylindrical	Titanium	3.6, 2.9, 3.6, 4.2, 5.0, 5.5, 6.0, 6.5	8, 10, 12, 14, 16, 18	von Mises MPa
Kheiralla et al. [46] 2014	Mandible	C + T	ND	ND	Titanium	3, 3.75, 5.7	8, 13	von Mises Mpa; μStrain
Kong et al. [47] 2008	Posterior mandible	C + T	II	Cylindrical	Titanium	2.5 to 5	6 to 16	von Mises MPa
Kong et al. [48] 2009	Posterior mandible	C + T	II	Cylindrical-screwed	ITI	3.0–5.0	6.0–16.0	Mpa
Kong et al. [49] 2009	Mandibular premolar	C + T	II	Cylinder screwed	ITI	3.0 to 5.0	6.0 to 16.0	von Mises MPa
Li et al. [50] 2009	Posterior maxilla	C + T	IV	Screwed	Titanium	3 to 5	6 to 14	Mpa
Li et al. [51] 2011	Posterior mandible	C + T	IV	Screwed	Titanium	3.0 to 5.0	6.0 to 16.0	Mpa
Moriwaki et al. [52] 2016	Posterior maxilla	C + T	ND	Threaded	Titanium	4, 5	6, 13	Mpa
Niroomand et al. [53] 2019	Posterior mandible	C + T	II	Threaded	Titanium	3.4, 4.1, 4.8	10, 13, 16	von Mises MPa
Niroomand et al. [54] 2020	Mandible	C + T	II	Threaded	Titanium	3.4, 4.1, 4.8	10, 13, 16	MPa
Özil et al. [55] 2023	Maxilla	C + T	II	Tapered	Titanium	4.1, 4.8	4, 4.1, 4.8,12, 14	von Mises Mpa
Park et al. [56] 2022	Posterior mandible	C + T	III, IV	Threaded	Ti–6Al–4V	4, 4.5, 5	7, 10, 13	MPa; μStrain
Pellizzer et al. [57] 2013	Posterior mandible	C + T	III	Cylinder screwed	Titanium	3.75, 5	10	von Mises MPa
Petrie et al. [58] 2005	Posterior mandible	C + T	II	Cylindrical or tapered	Titanium	3.5 to 6	5.75 to 23.5	μStrain
Porrua et al. [59] 2020	Mandibular premolar	C + T	II	Threaded	Ti–6Al–4V	3.8 to 4.5	10 to 13	Mpa
Raaj et al. [60] 2019	Posterior mandible	C + T	ND	Tapered	Titanium	3.5, 4.3	10, 11.5	Mpa
Sheikhan et al. [61] 2020	Mandible	C + T	ND	Threaded	Titanium	3.5, 4.5, 5.5	8, 10, 12	μStrain
Shinya et al. [62] 2021	Mandible	C + T	ND	Threaded	Titanium	3.8, 2.3, 6.0	9, 11, 13, 16	Mpa
Ueda et al. [63] 2016	Posterior mandible	C + T	III	Threaded	Titanium	3.5 to 6	8, 10, 11,13	Gpa
Vairo et al. [64] 2013	Maxilla premolar	C + T	II	Threaded	Ti–6Al–4V	3.5, 3.6, 4.3	5.5, 9, 11	von Mises MPa

Author (year)	Type of loading	Axial load	Oblique load	Lateral load	Experimental validation	Convergence analysis	Boundary conditions
Alqahtani et al. [25] 2023	Static	100 N	100 N; 45°	NA	NA	NA	Boundary conditions were established by constraining all nodes at the base of the 3D models.
Anitua et al. [26] 2010	Static	NA	150 N; 30°	NA	NA	NA	The external borders of the modeled bone section were constrained so that the displacement of the nodes in all directions was equal to 0.
Baggi et al. [27] 2008	Static	250 N	NA	100 N	NA	Y	Since the free length of bone segments (the distance between end surfaces of anatomical sites and the implant location) was sufficiently larger than the maximum dimension of the implant and in agreement with the theory of elasticity.
Balkaya et al. [28] 2014	Static	300 N	NA	NA	NA	NA	The bottom of the mandible was restrained against movement in the x, y, and z directions.
Bayrak et al. [29] 2020	Static	NA	200 N; 45°	NA	NA	NA	NA
Borie et al. [30] 2016	Static	NA	150 N; 45°	NA	NA	NA	NA
Bourauel et al. [31] 2012	Immediate	NA	150 N; 300 N; 30°	NA	NA	NA	The end faces of the idealised bone models were constrained in all three degrees of freedom.
Chakraborty et al. [32] 2022	Static	75 N	NA	NA	NA	Y	The connection between the implants and framework was considered to be completely bonded to avoid error due to relative micromovement between the implants and the framework.
Demenko et al. [33] 2014	Static	114.6 N	NA	17.1 N; 23.4 N	NA	NA	Nodes at both ends of the mandibular segment were restrained.
Demenko et al. [34] 2019	Static	114.6 N	NA	17.1 N; 23.4 N	NA	Y	NA
Ding et al. [35] 2009	Immediate	150 N	150 N; 45°	NA	NA	NA	Boundary conditions included constraining all three degrees of freedom at each of the nodes located at the joint surface of the condyles and the attachment regions of the masticatory muscles.
Ding et al. [36] 2009	Immediate	150 N	150 N; 45°	NA	NA	NA	Boundary conditions included constraining all three degrees of freedom at each of the nodes located in the front bevel face of the condyles.
Eazhil et al. [37] 2016	Static	114.6 N	NA	17.1 N; 23.4 N	NA	NA	NA
Elleuch et al. [38] 2021	Static	100 N	NA	17 N	NA	Y	The boundary conditions were prescribed to the side edges of the model, to restrict the translational and rotational displacements.
Faegh et al. [39] 2010	Static	113 N	NA	NA	NA	NA	Bone was restricted in all degrees of freedom along the inferior periphery.
Forna et al. [40] 2020	Static	114.6 N	NA	17.1 N; 23.4 N	NA	NA	Boundary conditions were applied to end surfaces of the mandibular model, fixed in all directions.
Georgiopoulos et al. [41] 2007	Immediate	118.2 N	NA	NA	NA	NA	Constraints were applied at the outer surface of the bone in order to prevent free body motion.
Guan et al. [42] 2010	Static	NA	NA	NA	NA	NA	When the slice was subjected to in-plane (x-y) masticatory forces (resulting from horizontal and vertical loading), it was restrained from deforming out of plane (in the z-axis).
Gümrükçü et al. [43] 2018	Static	150 N	NA	NA	NA	Y	The boundary condition was determined as the area from where the maxilla connected to the cranial base.
Güzelce et al. [44] 2023	Static	50 N	NA	NA	NA	NA	The models were fixed by restricting all degrees of freedom from the nodal points in the lower regions of the cortical bone and mucosa, preventing movement in all three axes.
Himmlová et al. [45] 2004	Static	114.6 N	NA	17.1 N; 23.4 N	NA	NA	The mesial and distal borders of the end of the modeled section of the mandible were constrained so that the displacement of nodes in all directions was equal to zero.
Kheiralla et al. [46] 2014	Static	300 N	NA	NA	Y	NA	The only restraint applied was a fixed restraint on the inferior surface of the mandible (the bottom surface), so no translation was allowed for this surface in all directions.
Kong et al. [47] 2008	Static	200 N	NA	100 N	NA	NA	Models were constrained in all directions at the nodes on the mesial and distal bones.
Kong et al. [48] 2009	Immediate	100 N	30 N; 45°	NA	NA	Y	Models were constrained in all directions at the nodes on the mesial and distal bone surfaces.
Kong et al. [49] 2009	Static	200 N	NA	100 N	NA	Y	Models were constrained in all directions at the nodes on the mesial and distal bone surfaces.
Li et al. [50] 2009	Static	100 N	30 N; 45°	NA	NA	Y	NA
Li et al. [51] 2011	Static	100 N	30 N; 45°	NA	NA	Y	The models were constrained at the nodes on the mesial and distal bones in all directions.
Moriwaki et al. [52] 2016	Static	NA	150 N; 30°	NA	NA	NA	NA
Niroomand et al. [53] 2019	Static	NA	100 N; 45°	NA	NA	NA	The distal and mesial regions are constrained with fixed boundary conditions.
Niroomand et al. [54] 2020	Static	NA	100 N; 45°	NA	NA	NA	Distal and mesial sides are fixedly supported.
Özil et al. [55] 2023	Static	100 N	NA	NA	NA	NA	Posterior region of the mandibular model was considered the boundary conditions and fixed in Degrees of Freedom (DOF) to be immobile in 3 axes.
Park et al. [56] 2022	Static	50 N	50 N; 30°	NA	NA	Y	As the boundary condition, the distal and mesial planes of the bone segment were fixed in all directions (X, Y, and Z).
Pellizzer et al. [57] 2013	Static	200 N	NA	NA	NA	NA	Boundary conditions were established as prescribed in the 3 axes (x, y, and z) on the side surfaces of cortical and trabecular bone, with the rest of the set free from restrictions.
Petrie et al. [58] 2005	Static	100 N	NA	20 N	NA	Y	Boundary conditions included constraining all three degrees of freedom at each of the nodes located at the most external mesial or distal aspect of the model.
Porrua et al. [59] 2020	Static	114.6 N	NA	17.1 N; 23.4 N	NA	NA	The boundary conditions included constraining all three degrees of freedom at x, y, and z directions (cortical and trabecular bones).
Raaj et al. [60] 2019	Static	100 N	NA	50 N; 50 N	NA	NA	NA
Sheikhan et al. [61] 2020	Static	100 N	NA	20 N	NA	NA	The fixed support boundary condition was applied to the bottom of the bone block. The frictionless support boundary condition was applied to the mesial and distal walls of the bone block.
Shinya et al. [62] 2021	Static	NA	50 N; 45°	NA	NA	NA	With regard to restriction conditions, the inferior border of the mandible was assumed to be completely fixed.
Ueda et al. [63] 2016	Static	60 N	60 N; 15°	NA	NA	NA	The nodes on the mesial and distal sections of the mandible were restrained in all directions.
Vairo et al. [64] 2013	Static	250 N	NA	100 N	NA	NA	All displacement degrees of freedom were prevented for any boundary node lying on the coronal sections delimiting the bone submodel.

C: cortical bone; T: trabecular bone; MPa: Megapascals; GPa: Gigapascals; NA: no applicable; Y: yes.

Table 4 summarizes the main findings of the 40 studies. Most studies utilized three components of the FEA model, which are trabecular bone, cortical bone, and implant. Abutment and superstructure were also considered in certain studies. Regarding the stability factor, the models were generally fixed at the bottom and sides of the bone to ensure zero movement in the degree of freedom, and all the components and the bone were usually assumed to be perfectly bonded. The literature also suggests other factors that may influence peri-implant stress, such as bone characteristics, implant system, cantilever length, thread features, and abutment collar height. Implant diameter and length mainly influence the stress distribution in cortical and cancellous bone, respectively. The diameter of dental implants is more important than implant length in reducing bone stress distribution and improving implant stability under the FEA model. The diameter of dental implants is considered to have an impact in minimizing peri-implant stress concentrations to avoid crestal overloading. In terms of stress reduction, the length of dental implant gains increasing relevance with reducing bone density. Short implants with a larger diameter were found to generate lower stresses than longer implants with a smaller diameter. Due to the results of different implant lengths and diameters in FEA models, no numerical answer could be concluded in terms of what peri-implant stress distribution increases, and it remains unclear what interaction between the diameter and length of dental implants exists.

**Table 4:** Summary of findings of included study.

Author (year)	Parts of FEM	**Stability factor**	Variables	Main findings
Alqahtani et al. [25] 2023	Implant, bone	NA	Implant diameter	For the treatment of atrophic ridges or in scenarios necessitating extensive surgical preparation of the implant site, a combination of short implants, wide diameters, and platform switching can be employed.
Anitua et al. [26] 2010	Bone, implant-abutment	The implant was considered to be perfectly osseointegrated.	Implant diameter, implant length	Using wider implants may be better to dissipate the acting forces and reduce stress in the bone surrounding the implant. The use of shorter and wider implants might be a reasonable alternative in sites limited by the height of the residual ridge.
Baggi et al. [27] 2008	Maxillary and mandibular bone; cortical bone, trabecular bone, the cancellous bone-implant interface	All nodal displacement components of segments were set equal to zero.	Maxillary and mandibular bone; type of implant; implant total length; bone-implant interface length; implant maximum diameter; average thread pitch; average thread depth.	Cortical peri-implant areas that could be affected by overloading were influenced primarily by implant diameter. An increase in implant length reduced stress gradients at the cancellous peri-implant region.
Balkaya et al. [28] 2014	Implant, framework, cancellous bones, cortical bones	Fully bonded interaction was modeled along the implant-bone interface to simulate a completely osseointegrated implant that directly bonded to the surrounding bone.	Varying number, inclination, and sizes of implants	Short implants with large-diameter showed lower stresses than long implants with small diameter. Increasing diameter may decrease high stress concentration from increasing cantilever length.
Bayrak et al. [29] 2020	Cancellous bone, cortical bone, implant,	The models were fixed at the bottom and sides of the bone so that they had zero movement in the degree of freedom (DOF).	Implant diameter, implant length, type of implant	The triple cylindrical implants, with a new implant design, showed appropriate results in terms of abutment, implant, and bone tissue stress.
Borie et al. [30] 2016	Implants, abutments and frameworks	All abutments were fixed to the implant based on a perfect adaptation and a complete joint. The implant was considered to be completely osseointegrated.	Implant lengths, connections, locations, and restoration materials	The implant connection system, length, restoration material, and type of prosthesis influence the stresses at the peri-implant bone. Implants of 10 mm in length exhibited higher stress values.
Bourauel et al. [31] 2012	Cancellous bone, cortical bone, implant	The studied implants were inserted into idealised bone segments.	Implant diameter, length	Implant diameter and geometry had a pronounced effect on stresses in the cortical plate. Stress in spongy and cortical bone around short implants were markedly increased compared to those in standard implants.
Chakraborty et al. [32] 2022	Implants, framework and bone	The temporomandibular joint was assumed to be fixed in all the directions.	Implant–bone interface condition, implant and framework design factors	Implant diameter had more effect compared to implant length toward peri-implant bone biomechanical response.
Demenko et al. [33] 2014	Cancellous bone, cortical bone, implant	Implants were assumed to be completely osseointegrated and placed at the midspan of the segment	Implant diameter, length	There exists a certain spectrum of diameter-to-length ratios, which will keep maximum bone stresses at a preset level chosen based on patient's bone strength.
Demenko et al. [34] 2019	Cancellous bone, cortical bone, implant,	NA	Implant diameter, length	Implant load-carrying capacity depends on diameter and available bone height. Wide implants have higher load-carrying capacity than narrow implants. Short implants with proper diameter and length avoid bone overstress, even in Type IV bone.
Ding et al. [35] 2009	Cancellous bone, cortical bone, implant,	The implant–bone interface was assumed as before the occurrence of osseointegration and simulated by nonlinear contact zones with friction.	Implant diameter, length	Increasing the diameter and length of the implant decreased the stress and strain on the alveolar crest, and the stress and strain values notably increased under buccolingual loading as compared with vertical loading, but diameter had a more significant effect than length to relieve the crestal stress and strain concentration.
Ding et al. [36] 2009	Cancellous bone, cortical bone, implant,	It was modeled using nonlinear frictional contact elements, which allowed minor displacements between implant and bone.	Diameter of implant	With an increase of implant diameter, stress and strain on the implant– bone interfaces significantly decreased, especially when the diameter increased from 3.3 to 4.1 mm.
Eazhil et al. [37] 2016	Cancellous bone, cortical bone, implant,	Implants were estimated to be completely osseointegrated.	Implant diameter, length	There was statistically significant decrease in von Mises stress as the implant diameter increased.
Elleuch et al. [38] 2021	Jaw bones, implant and abutment	The interfaces between the native teeth, the cortical and cancellous bones are treated as perfect bonding.	Diameter, length and thread's pitch	The implant diameter is identified to be the dominant variable. The maximum equivalent stresses in the abutment, implant, and jaw bones decrease considerably with the increase of the implant diameter.
Faegh et al. [39] 2010	Trabecular bone, cortical bone, implant	All the components and the bone were assumed to be perfectly bonded.	General contour, external threads	The slope and length of the implant collar, and the implant diameter influence the interfacial stress levels the most, and the effects of changing these parameters are significantly noticed only in the cortical bone area.
Forna et al. [40] 2020	Implant, abutment, bone	The contact type between bone and implant was defined to be perfectly bonded.	Implant diameter, length and type of bone	Diameter and length play an equally important role in decreasing stress.
Georgiopoulos et al. [41] 2007	Cortical bone, Trabecular bone, Dental implant & abutment, Superstructure	The contact surfaces of implant and surrounding bone were always bonded, with no sliding permitted (fixed bond).	Implant diameter, length	The FEA results indicated a tendency towards stress reduction on the implant when the length was increased. As far as bone tissue was concerned, there was a tendency towards strain reduction when the length of the implant was increased from 10 mm up to 14 mm.
Guan et al. [42] 2010	Cancellous bone, cortical bone, implant,	Fifty Percent Osseointegration Between Implant and Bone The interface surrounding an implant includes both blood and bone fragments.	Implant diameter, length, Young's modulus of cancellous bone, Young's modulus of cortical bone, the cortical bone thickness	The implant length is more influential within cancellous bone than the diameter. However, implant diameter is more influential in cortical bone.
Gümrükçü et al. [43] 2018	Cancellous bone and cortical bone	We assumed that there was excellent osseointegration in bone-implant interface in all models.	Implant number, length and tilting degree	The ideal implant length is 11.5 mm.
Güzelce et al. [44] 2023	Cancellous bone, cortical bone, implant, Abutment Screw Crown Temporary cement	The denture and implant were provided with bonded contact for all models.	Implant diameter, framework materials	Mini-implants produce signifcantly higher stress values in the supporting tissues and implant neck than standard implants.
Himmlová et al. [45] 2004	Bone and implant	The interface between the implant and the bone was modeled as an immovable junction.	Implant diameter, length	An increase in the implant diameter decreased the maximum von Mises equivalent stress around the implant neck more than an increase in the implant length, as a result of a more favorable distribution of the simulated masticatory forces applied in this study.
Kheiralla et al. [46] 2014	Trabecular bone, cortical bone, implant	All components were constructed in a way that ensures 100% contact along interfaces with no gaps or interferences.	Size of implant, loading conditions	Standard and short-wide implants could be a better choice than narrow implants in supporting single-unit restorations.
Kong et al. [47] 2008	Cortical bone, Cancellous bone	The implant was rigidly anchored in the bone model along its entire interface.	Implant diameter, length	Implant diameter and length favor stress distribution in cortical bone and cancellous bone, respectively. Implant diameter exceeding 3.9 mm and implant length exceeding 10.0 mm are the optimal choice for type B/2 bone in a cylinder implant. The implant diameter is more important than length in reducing bone stress.
Kong et al. [48] 2009	Cancellous bone, cortical bone, implant,	The prosthesis– abutment interface was considered to be bonded.	Implant diameter, length	Exceeding 4.0 mm and longer 11.0 mm are the best combination for optimal biomechanical properties in immediate loading implants in the type B/2 bone.
Kong et al. [49] 2009	Cancellous bone, cortical bone, and implant-abutment	For simulation, a ‘‘fixed bond” condition was set to its interface with the bone.	Implant diameter, length	The implant diameter affected stress distribution in jaw bone more than length did; and an implant diameter exceeding 3.9 mm and implant length exceeding 9.5 mm was the optimal selection for type B/2 bone in a cylinder implant by biomechanical considerations.
Li et al. [50] 2009	Cortical and cancellous bones, implant–abutment complex	A bond condition was set at its interface with the bone.	Implant diameter, length	In type IV bone, implant length is more crucial in reducing bone stress and enhancing the stability of implant-abutment complex than implant diameter. Biomechanically, implant diameter exceeding 4.0 mm and implant length exceeding 9.0 mm are the combination with optimal properties for a screwed implant in type IV bone.
Li et al. [51] 2011	Cancellous bone, cortical bone, implant	During the simulation, a bond condition was set at its interface with the mandibular bone.	Implant diameter, length	In the posterior mandible, implant diameter plays more significant roles than length in reducing cortical bone stress and enhancing implant stability under both loads. Meanwhile, implant length is more effective than diameter in reducing cancellous bone stress under both loads. Moreover, biomechanically, implant diameter exceeding 4.0 mm and implant length exceeding 12.0 mm is a relatively optimal combination for a screwed implant in the posterior mandible with poor bone quality.
Moriwaki et al. [52] 2016	Cortical bone, Cancellous bone, Implant components, Graft material	For simulations of osseointegrated implants, a “fixed bond” condition was set at the interface between the bone or graft material and the implant body.	Implant diameter, length	4-mm-diameter implants with increased length should be selected to reduce the maximum principal stress of peri-implant cortical bone when bone quantity A is available. When there is bone quantity C, 6-mm-length implants should be selected if the bone width is sufficient to permit increasing the implant diameter from 4.0 mm to 5.0 mm.
Niroomand et al. [53] 2019	Implant–abutment Cancellous bone	To have neither separation nor sliding between implant and mandible section, the bonded type of condition is chosen for their interface. The contact type between cancellous and cortical bones is bonded as well.	Implant diameter, length and thread depth, width, inner angle, and pitch	The implant length is the most effective factor in reducing the stress in implant–abutment while it shows no significant effect on the magnitude of stress in cancellous bone. The implant length is the most effective factor in the magnitude of Max von Mises stress in implant–abutment, while the implant diameter has a significant effect the Max von-Mises stress in cancellous bone.
Niroomand et al. [54] 2020	Implant, abutment, cancellous bones, cortical bones	The bonded type of interface condition is used between implant and mandible.	The implant length and diameter together with thread depth, width, pitch, and inner angle	Increasing the diameter of implant leads to reducing the amount of von Mises stress in crestal area of cortical bone. Moreover, the increase of length enhances the contact between implant and cancellous bone by which the displacement of cancellous is reduced.
Özil et al. [55] 2023	Bone, implant, abutment	The implant-bone interface was considered to be completely osseointegrated.	Implant diameter, length	Short implant placement in the posterior region in the all-on-four concept reduces stress on the bone, implants, and prosthetic parts, regardless of the diameter of the short implant.
Park et al. [56] 2022	Cancellous bone, cortical bone, implant, Abutment Screw Crown Temporary cement	All surfaces with implant complex-to-bone and bone-to-bone contacts were assumed to be in tie condition.	Implant design factors, bone quality	For implants of shorter length or narrower diameter, the volume fraction in the range of fatigue failure was large regardless of the initial bone condition. the need for performing the FEA considering the bone remodeling process is increased when placing a short-length and narrow-diameter implant on a poor-quality bone.
Pellizzer et al. [57] 2013	Cancellous bone, cortical bone and implant	The contacts between the prosthetic component/screw, implant/cortical bone, implant/trabecular bone, cortical/trabecular bone, and implant/screw were assumed to be bonded.	Implant diameter, sizes of Hexagon	Among the models of wide diameter (models B and C), model B (implant 5.00 mm/regular hexagon) was more favorable with regard to distribution of stresses. Model A (implant 3.75 mm/regular hexagon) showed the largest areas and the most intense stress, and model B (implant 5.00 mm/regular hexagon) showed a more favorable stress distribution. The highest stresses were observed in the application of lateral load.
Petrie et al. [58] 2005	High-density cancellous bone, Low-density cancellous bone, Cortical bone	We assumed complete or 100% osseointegration at the implant/bone interface and we modeled the restoration and abutment as a seamless/continuous unit.	Diameter, length of tapered segment, length of untapered segment, and taper	If the objective is to minimize peri-implant strain in the crestal alveolar bone, a wide and relatively long implant appears to be the most favorable choice. Narrow, short implants with taper in the crestal region should be avoided, especially in low density bone.
Porrua et al. [59] 2020	Cancellous bone, cortical bone, implant,	NA	Implant diameter, length, and elastic modulus	The interactions of the diameter of the implant with its length and its elastic modulus have a statistically significant influence on the von Mises equivalent stress values. The implant diameter, the implant length, and its interaction showed statistically significant influence on the von Mises stress in the peri-implant trabecular bone.
Raaj et al. [60] 2019	Cortical bone, cancellous bone, implant	NA	Implant diameter, implant length	In axial and non-axial loads, amount of stress distribution around implant–bone interface is influenced by diameter and length of implant in cortical and cancellous bone, respectively. Increased diameter of the implant produces the minimum stress in cortical bone. Increased length of the implant produces the minimum stress in cancellous bone.
Sheikhan et al. [61] 2020	Cancellous bone, cortical bone, implant,	The bonded condition was applied between the abutment and the implant, and also at the bone-implant interface.	Implant length, diameter, and taper, thread depth and thread angle	Compared with other parameters, the diameter is by far the most influential parameter on the peak compressive and tensile strains at cortical and cancellous interfaces.
Shinya et al. [62] 2021	Cortical bone, trabecular bone, implant	The space in the mandibular ridge following virtual extraction was set to be automatically replaced by bone, so there was no bone in the space when the implant was placed.	Implant diameter, length	The stress on the peri-implant bone was found to decrease with increasing length and mainly in diameter of the implant.
Ueda et al. [63] 2016	Trabecular bone, cortical bone, implant,	NA	Thickness of the cortical bone, Young's modulus of the trabecular bone, and the diameter and length of the implant	Implants of proper length or diameter could limit the maximum equivalent strain in peri-implant bone except when both the thickness of the cortical bone and the Young's modulus of the trabecular bone are small.
Vairo et al. [64] 2013	Cancellous bone, cortical bone, implant	Complete osseous integration between implant and bone tissue was assumed.	Implant design, in-bone positioning depth, and bone posthealing crestal morphology	The implant diameter can be retained as a more effective design parameter than the implant length. A significant reduction of stress peaks, mainly at the cortical bone, occurred when implant diameter increased. Nevertheless, implant length exhibited a certain influence on the bone-implant mechanical interaction at the cancellous interface, resulting in more effective and homogeneous stress distributions in trabecular bone when the implant length increased.

NA: no applicable.

### **3.3** Quality assessment

A total of 40 studies were evaluated and ranked based on the information presented in Table 5. In general, most of the studies utilized complex or very complex bone models. However, when it comes to the design of the implant, only 23 of the selected studies employed commercially available implant designs, which limited the scope of the research. While 23 studies reported loading in multiple directions, the remaining 17 studies only applied axial load. The majority of studies featured more than 100,000 elements, with only 2 studies using two-dimensional FEA. As for prosthetic restoration, 16 studies utilized crowns or bridges as a superstructure to enhance the FEA model's reliability. Due to the unavailability of meta-analysis data such as means, standard deviations, and sample size in FEA studies, a systematic synthesis approach was adopted based on the research questions proposed to thematically explore the results and methods.

**Table 5:** Total scores of included studies.

Author (year)	Implant model	Bone model	Element number	Type of loading	Prosthetic restoration	Dimensions	Score
Alqahtani et al. [25] 2023	Poor	Complex	<50,000	Multiple directions	Crown/bridge	3D	8
Anitua et al. [26] 2010	Very complex	Poor	<100,000	Axial	–	3D	8
Baggi et al. [27] 2008	Very complex	Very complex	>100,000	Multiple directions	–	3D	12
Balkaya et al. [28] 2014	Complex	Complex	>100,000	Axial	–	3D	9
Bayrak et al. [29] 2020	Poor	Complex	>100,000	Axial	Crown/bridge	3D	9
Borie et al. [30] 2016	Complex	Very complex	>100,000	Axial	Crown/bridge	3D	11
Bourauel et al. [31] 2012	Very complex	Very complex	>100,000	Axial	–	3D	11
Chakraborty et al. [32] 2022	Poor	Very complex	>100,000	Axial	–	3D	9
Demenko et al. [33] 2014	Poor	Very complex	>100,000	Multiple directions	–	3D	10
Demenko et al. [34] 2019	Poor	Complex	>100,000	Multiple directions	–	3D	9
Ding et al. [35] 2009	Complex	Very complex	–	Multiple directions	–	3D	8
Ding et al. [36] 2009	Complex	Very complex	<50,000	Multiple directions	–	3D	9
Eazhil et al. [37] 2016	Complex	Complex	<50,000	Multiple directions	–	3D	8
Elleuch et al. [38] 2021	Poor	Complex	–	Multiple directions	–	3D	6
Faegh et al. [39] 2010	Poor	Very complex	>100,000	Axial	–	3D	9
Forna et al. [40] 2020	Poor	Very complex	–	Multiple directions	–	3D	7
Georgiopoulos et al. [41] 2007	Poor	Complex	<50,000	Axial	Crown/bridge	2D	6
Guan et al. [42] 2010	Poor	Complex	<50,000	Multiple directions	–	2D	6
Gümrükçü et al. [43] 2018	Complex	Very complex	–	Axial	Crown/bridge	3D	8
Güzelce et al. [44] 2023	Complex	Very complex	>100,000	Axial	Crown/bridge	3D	11
Himmlová et al. [45] 2004	Complex	Poor	<50,000	Multiple directions	–	3D	6
Kheiralla et al. [46] 2014	Complex	Complex	–	Axial	Crown/bridge	3D	7
Kong et al. [47] 2008	Poor	Complex	<50,000	Multiple directions	Crown/bridge	3D	8
Kong et al. [48] 2009	Complex	Complex	>100,000	Multiple directions	Crown/bridge	3D	10
Kong et al. [49] 2009	Complex	Very complex	<50,000	Multiple directions	–	3D	9
Li et al. [50] 2009	Complex	Complex	>100,000	Multiple directions	Crown/bridge	3D	10
Li et al. [51] 2011	Complex	Very complex	>100,000	Multiple directions	Crown/bridge	3D	11
Moriwaki et al. [52] 2016	Poor	Complex	>100,000	Axial	–	3D	8
Niroomand et al. [53] 2019	Poor	Complex	>100,000	Axial	–	3D	8
Niroomand et al. [54] 2020	Complex	Complex	>100,000	Axial	Crown/bridge	3D	10
Özil et al. [55] 2023	Complex	Very complex	>100,000	Axial	Crown/bridge	3D	11
Park et al. [56] 2022	Complex	Very complex	>100,000	Multiple directions	Crown/bridge	3D	11
Pellizzer et al. [57] 2013	Complex	Very complex	–	Axial	–	3D	7
Petrie et al. [58] 2005	Poor	Complex	<50,000	Multiple directions	Crown/bridge	3D	7
Porrua et al. [59] 2020	Poor	Complex	>50,000	Multiple directions	–	3D	8
Raaj et al. [60] 2019	Poor	Very complex	–	Multiple directions	–	3D	6
Sheikhan et al. [61] 2020	Poor	Complex	>100,000	Multiple directions	–	3D	10
Shinya et al. [62] 2021	Complex	Very complex	–	Axial	–	3D	7
Ueda et al. [63] 2016	Complex	Complex	–	Multiple directions	Crown/bridge	3D	7
Vairo et al. [64] 2013	Complex	Very complex	–	Multiple directions	–	3D	8

## 4 Discussion

This study aimed to assess the impact of both the diameter and length of dental implants on biomechanical properties using FEA. The included studies have reached a noteworthy conclusion that both implant diameter and length have a significant influence on the stress distribution of both cortical and cancellous bone under both static and immediate loading on dental implants or prosthetics. The findings of this study suggest that the diameter of dental implants is more important than the implant length in reducing bone stress distribution and improving implant stability under both static and immediate loading conditions, which is in accordance with the hypothesis of this study.

Implant diameter is one of the most critical parameters in dental implant design, as it significantly impacts the stress distribution around the implant-bone interface, particularly in cortical bone. Studies have indicated that implant diameter primarily affects the cortical peri-implant regions, with stress peaks of the cortical bone decreasing as the implant diameter increases [27]. In addition, when placing wider dental implants in the bone, a significant reduction of tensile and compressive stress values was observed. Eazhil et al. reported a significant reduction in von Mises stress with an increase in implant diameter [37]. Furthermore, increasing implant diameter can resolve the high-stress concentration caused by increasing cantilever length [28]. However, it should be noted that in low-density bone, the use of narrow-diameter implants with a taper in the crestal region must be avoided to ensure safety.

The length of a dental implant is the most influential factor in determining the magnitude of Max von Mises stress in the implant-abutment connection, with longer implants promoting more even stress distribution in trabecular bone compared to shorter implants. According to studies, implant length is a more crucial parameter than diameter in reducing cancellous bone stress under both axial and buccolingual loads [51]. With the increase in implant length, a decreased tendency towards peri-implant stress may occur, resulting in more effective and homogeneous stress distributions in trabecular bone [64]. When short implants are used, stresses in cancellous bone and strains in cortical bone increase significantly compared to standard implants [31]. The values of strains obtained from short implants were significantly higher compared to long implants, which exceeded the limitations of strains in the cancellous bone. However, Demenko et al. proposed that short implants with an appropriate length and diameter could avoid overstress in surrounding bone, even in low-quality bone [30]. In some cases where there is insufficient bone quantity, an implant length of 6 mm can be used if the bone width is sufficient [51].

A previous meta-analysis compared the survival rate of standard-diameter implants and narrow-diameter implants and indicated guidelines and recommendations for the application of narrow-diameter implants [7]. Another systematic review evaluated short implants concerning biomechanical properties and detected the most relevant parameters using FEA [24]. Accordingly, the main goal of the present review was to conduct a comprehensive assessment of the influence of both the diameter and length of dental implants. While the implant length presents an impact on stress distribution, the diameter is considered the most significant variable affecting stress distribution in the implant, abutment, and bone [37]. Some researchers have suggested that both diameter and length play an equal role in stress reduction [40]. However, most of the included studies suggest that diameter has a greater effect than length in reducing cortical bone stress and increasing implant stability under both static and immediate load [32,35,47,51]. For single crown restoration, Kheiralla et al. found that short implants were better than narrow-diameter implants, and another study found that short implants with a large diameter had lower stresses than long implants with a small diameter, supporting Kheiralla et al.'s conclusion [28,46]. After inserting 12 different implant diameters and lengths based on a CBCT model of the mandible, Shinya et al. concluded that stress distribution on surrounding bone was found to reduce with increasing length but mainly in implant diameter [62]. Therefore, to minimize the risk of overloading and improve implant stability, the diameter of the dental implant should be considered a more important parameter than implant length during the design process.

The longevity of implants relies on both endogenous and exogenous factors. Apart from exogenous factors such as the diameter and length of implants, endogenous factors including bone quality and quantity are also essential for the success of implantation. Type IV bone is defined as a thin layer of cortical bone surrounding trabecular bone with poor strength and low density [50]. This bone quality is typically found in the posterior maxilla, which is the primary area for tooth loss and the main region for masticatory activity. Accordingly, it is critical to investigate the role of implant diameter and length in these regions. However, few studies in this review examined the impact of implants with various lengths and diameters on poor-quality bone. One such study concluded that a screwed implant with a length of 12 mm and a diameter of 4 mm is the optimal combination for the posterior region of lower teeth with low density [51]. In addition, Li et al. concluded that dental implants with a diameter of 4 mm and a length of 9 mm were the best choice for a screwed implant in Type IV bone [50]. Therefore, evaluating the stability of short and narrow-diameter implants is essential under the conditions of placing these implants in Type IV bones.

The success of dental implantation depends on various factors, including implant diameter, implant length, bone quality, and other design factors such as thread features, implant system, and abutment collar height. Improving bone quality reduces bone strain values, and implants with 10 to 20-degree neck configurations are recommended to reduce strain values and enhance load dissipation in bone tissue [37,65,66]. The implant connection system, type of prosthesis, and restoration material can influence stresses in peri-implant bone, and the thread features, length, and slope of the implant collar are other factors to consider. Distal cantilevers may cause high strain on the cervical cortical bone, which can be addressed by increasing implant diameter [28,32]. Short implants exhibit the highest stress concentrations around the first threads for the screw, whereas long implants exhibit the highest von Mises stress at their neck [67,68]. Small dental implants have stress concentrations at the threads in the cervical and middle regions, and trapezoid-shaped threads are preferable over saw-tooth threads for inducing compressive and tensile states at the cortical bone. Conical connection and switching platform with a dental implant system present lower maximum strains around peri-implant areas, and longer abutment collars concentrate stresses at both cortical bone and implant levels through enhancing crown-to-implant ratios [69,70]. In summary, these potential influential design factors should be considered during clinical practice in implantology.

The loading condition is an essential part of the FEA, applying loadings from multiple directions can achieve a more reliable result. However, nearly half of the included studies only applied axial load, which may lead to limitations in the results. Accordingly, loading in multiple directions should be utilized in further studies to improve the reliability of the results of FEA. Boundary condition was also important in the FEA model. This condition was applied in most included studies as the models were generally fixed by restricting all degrees of freedom from the nodal point and preventing movement in all three axes. In addition, some researches also added a realistic approach to obtain the morphed geometry of the mandible and made the boundary condition more perceptible. Another important issue that needs to be considered is that only one study of this review conducted experimental validation for the FEA model and 11 researches used convergence analysis. Therefore, it is necessary to conduct experimental validation to confirm the results of the biomechanical evaluation of the length and diameter of dental implants with FEA.

This study has several limitations. Firstly, it is important to note that FEA models are simulations, and the accuracy of FEA models depends on the input data and assumptions made during the process of modeling. Therefore, results obtained from FEA models should be interpreted with caution and confirmed by in vivo and in vitro studies. Moreover, the present study only considered the effects of the length and diameter of dental implants on peri-implant stress distribution, while other factors, such as occlusal forces and implant placement techniques, were not considered. Future studies should take these factors into account for a more comprehensive understanding of implant biomechanics.

## 5 Conclusions

Based on the findings of this study, the following conclusions were drawn.

1. Implant diameter and length mainly influence the stress distribution in cortical and cancellous bone, respectively.
2. Implant diameter demonstrated a more significant effect compared to implant length in reducing bone stress distribution and enhancing implant stability.
3. Short implants with large diameters presented lower stresses than the long ones with small diameters.
4. Implant system, cantilever length, thread features, and abutment collar height should also be considered.

## Data availability statement

The data of this study will be available from the corresponding author on reasonable request.

## Funding statement

This research was funded by the 10.13039/501100012166National Key Research and Development Program of China, grant number 2021YFC2400400 and the 10.13039/501100001809National Natural Science Foundation of China, grant number 81400485.

## Additional information

No additional information is available for this paper.

## CRediT authorship contribution statement

**Piaopiao Qiu:** Visualization, Validation, Software, Investigation. **Rongkai Cao:** Writing – original draft, Methodology, Formal analysis, Conceptualization. **Zhaoyang Li:** Data curation. **Zhen Fan:** Writing – review & editing, Supervision, Resources, Project administration, Funding acquisition.

## Declaration of competing interest

The authors declare that they have no known competing financial interests or personal relationships that could have appeared to influence the work reported in this paper.

## References

[1] Buser D., Janner S.F., Wittneben J.G., Brägger U., Ramseier C.A., Salvi G.E. (2012). 10-year survival and success rates of 511 titanium implants with a sandblasted and acid-etched surface: a retrospective study in 303 partially edentulous patients. Clin. Implant Dent. Relat. Res..

[2] de Souza A.B., Sukekava F., Tolentino L., César-Neto J.B., Garcez-Filho J., Araújo M.G. (2018). Narrow- and regular-diameter implants in the posterior region of the jaws to support single crowns: a 3-year split-mouth randomized clinical trial. Clin. Oral Implants Res..

[3] Heydecke G., Locker D., Awad M.A., Lund J.P., Feine J.S. (2003). Oral and general health-related quality of life with conventional and implant dentures. Community Dent. Oral Epidemiol..

[4] Chiapasco M., Casentini P., Zaniboni M. (2009). Bone augmentation procedures in implant dentistry. Int. J. Oral Maxillofac. Implants.

[5] Jensen S.S., Terheyden H. (2009). Bone augmentation procedures in localized defects in the alveolar ridge: clinical results with different bone grafts and bone-substitute materials. Int. J. Oral Maxillofac. Implants.

[6] Doonquah L., Lodenquai R., Mitchell A.D. (2015). Surgical techniques for augmentation in the horizontally and vertically compromised alveolus. Dent. Clin..

[7] Schiegnitz E., Al-Nawas B. (2018). Narrow-diameter implants: a systematic review and meta-analysis. Clin. Oral Implants Res..

[8] Monje A., Chan H.L., Fu J.H., Suarez F., Galindo-Moreno P., Wang H.L. (2013). Are short dental implants (<10 mm) effective? a meta-analysis on prospective clinical trials. J. Periodontol..

[9] Allum S.R., Tomlinson R.A., Joshi R. (2008). The impact of loads on standard diameter, small diameter and mini implants: a comparative laboratory study. Clin. Oral Implants Res..

[10] Yamaguchi K., Ishiura Y., Tanaka S., Baba K. (2014). Influence of the rigidity of a provisional restoration supported on four immediately loaded implants in the edentulous maxilla on biomechanical bone-implant interactions under simulated bruxism conditions: a three-dimensional finite element analysis. Int. J. Prosthodont. (IJP).

[11] Pommer B., Hingsammer L., Haas R. (2015). Denture-Related biomechanical factors for fixed partial dentures retained on short dental implants. Int. J. Prosthodont. (IJP).

[12] Ouldyerou A., Merdji A., Aminallah L., Msomi V., Chong Pl P.L., Roy S. (2022). Biomechanical evaluation of marginal bone loss in the surrounding bone under different loading: 3d finite element analysis study. Int. J. Multiscale Comput. Eng..

[13] Panagiotopoulou O. (2009). Finite element analysis (FEA): applying an engineering method to functional morphology in anthropology and human biology. Ann. Hum. Biol..

[14] Huempfner-Hierl H., Schaller A., Hemprich A., Hierl T. (2014). Biomechanical investigation of naso-orbitoethmoid trauma by finite element analysis. Br. J. Oral Maxillofac. Surg..

[15] Trivedi S. (2014). Finite element analysis: a boon to dentistry. J Oral Biol Craniofac Res.

[16] Geng J.P., Tan K.B., Liu G.R. (2001). Application of finite element analysis in implant dentistry: a review of the literature. J. Prosthet. Dent.

[17] Campaner L.M., Silveira M.P.M., de Andrade G.S. (2021). Influence of polymeric restorative materials on the stress distribution in posterior fixed partial dentures: 3D finite element analysis. Polymers.

[18] Ausiello P., Dal Piva A., Borges A.L.S. (2021). Effect of shrinking and No shrinking dentine and enamel replacing materials in posterior restoration: a 3D-FEA study. Appl. Sci..

[19] Park J.H., Moon H.S., Jung H.I., Hwang J., Choi Y.H., Kim J.E. (2023). Deep learning and clustering approaches for dental implant size classification based on periapical radiographs. Sci. Rep..

[20] Rekawek P., Herbst E.A., Suri A., Ford B.P., Rajapakse C.S., Panchal N. (2023). Machine learning and artificial intelligence: a web-based implant failure and peri-implantitis prediction model for clinicians. Int. J. Oral Maxillofac. Implants.

[21] Jung R.E., Herzog M., Wolleb K., Ramel C.F., homa D.S., Hämmerle C.H. (2017). A randomized controlled clinical trial comparing small buccal dehiscence defects around dental implants treated with guided bone regeneration or left for spontaneous healing. Clin. Oral Implants Res..

[22] Moher D., Shamseer L., Clarke M. (2015). Preferred reporting items for systematic review and meta-analysis protocols (PRISMA-P) 2015 statement. Syst. Rev..

[23] Schardt C., Adams A.B., Owens T., Keitz S., Fontelo P. (2007). Utilization of the PICO framework to improve searching PubMed for clinical questions. BMC Med Inform Decis Mak.

[24] Hingsammer L., Pommer B., Hunger S., Stehrer R., Watzek G., Insua A. (2019). Influence of implant length and associated parameters upon biomechanical forces in finite element analyses: a systematic review. Implant Dent..

[25] Alqahtani A.R., Desai S.R., Patel J.R. (2023). Investigating the impact of diameters and thread designs on the Biomechanics of short implants placed in D4 bone: a 3D finite element analysis. BMC Oral Health.

[26] Anitua E., Tapia R., Luzuriaga F., Orive G. (2010). Influence of implant length, diameter, and geometry on stress distribution: a finite element analysis. Int. J. Periodontics Restor. Dent..

[27] Baggi L., Cappelloni I., Di Girolamo M., Maceri F., Vairo G. (2008). The influence of implant diameter and length on stress distribution of osseointegrated implants related to crestal bone geometry: a three-dimensional finite element analysis. J. Prosthet. Dent.

[28] Balkaya M.C. (2014). Investigation of influence of different implant size and placement on stress distribution with 3-dimensional finite element analysis. Implant Dent..

[29] Bayrak A., Yaramanoğlu P., Kılıçarslan M.A., Yaramanoğlu B., Akat B. (2020). Biomechanical comparison of a new triple cylindrical implant design and a conventional cylindrical implant design on the mandible by three-dimensional finite element analysis. Int. J. Oral Maxillofac. Implants.

[30] Borie E., Orsi I.A., Noritomi P.Y., Kemmoku D.T. (2016). Three-dimensional finite element analysis of the biomechanical behaviors of implants with different connections, lengths, and diameters placed in the maxillary anterior region. Int. J. Oral Maxillofac. Implants.

[31] Bourauel C., Aitlahrach M., Heinemann F., Hasan I. (2012). Biomechanical finite element analysis of small diameter and short dental implants: extensive study of commercial implants. Biomed. Tech..

[32] Chakraborty A., Datta P., Kumar C.S., Majumder S., Roychowdhury A. (2022). Probing combinational influence of design variables on bone biomechanical response around dental implant-supported fixed prosthesis. J. Biomed. Mater. Res. B Appl. Biomater..

[33] Demenko V., Linetskiy I., Nesvit K., Hubalkova H., Nesvit V., Shevchenko A. (2014). Importance of diameter-to-length ratio in selecting dental implants: a methodological finite element study. Comput. Methods Biomech. Biomed. Eng..

[34] Demenko V., Linetskiy I., Linetska L., Yefremov O. (2019). Load-carrying capacity of short implants in edentulous posterior maxilla: a finite element study. Med. Eng. Phys..

[35] Ding X., Liao S.H., Zhu X.H., Zhang X.H., Zhang L. (2009). Effect of diameter and length on stress distribution of the alveolar crest around immediate loading implants. Clin. Implant Dent. Relat. Res..

[36] Ding X., Zhu X.H., Liao S.H., Zhang X.H., Chen H. (2009). Implant-bone interface stress distribution in immediately loaded implants of different diameters: a three-dimensional finite element analysis. J. Prosthodont..

[37] Eazhil R., Swaminathan S.V., Gunaseelan M., Kannan G.V., Alagesan C. (2016). Impact of implant diameter and length on stress distribution in osseointegrated implants: a 3D FEA study. J. Int. Soc. Prev. Community Dent..

[38] Elleuch S., Jrad H., Kessentini A., Wali M., Dammak F. (2021). Design optimization of implant geometrical characteristics enhancing primary stability using FEA of stress distribution around dental prosthesis. Comput. Methods Biomech. Biomed. Eng..

[39] Faegh S., Müftü S. (2010). Load transfer along the bone-dental implant interface. J. Biomech..

[40] Forna D.A., Forna N.C., Butnaru Moldoveanu S.A. (2020). Influence of implant dimensions in the resorbed and bone augmented mandible: a finite element study. Contemp. Clin. Dent..

[41] Georgiopoulos B., Kalioras K., Provatidis C., Manda M., Koidis P. (2007). The effects of implant length and diameter prior to and after osseointegration: a 2-D finite element analysis. J. Oral Implantol..

[42] Guan H., van Staden R., Loo Y.C., Johnson N., Ivanovski S., Meredith N. (2010). Evaluation of multiple implant-bone parameters on stress characteristics in the mandible under traumatic loading conditions. Int. J. Oral Maxillofac. Implants.

[43] Gümrükçü Z., Korkmaz Y.T. (2018). Influence of implant number, length, and tilting degree on stress distribution in atrophic maxilla: a finite element study. Med. Biol. Eng. Comput..

[44] Güzelce S.E. (2023). Biomechanical comparison of different framework materials in mandibular overdenture prosthesis supported with implants of different sizes: a finite element analysis. BMC Oral Health.

[45] Himmlová L., Dostálová T., Kácovský A., Konvicková S. (2004). Influence of implant length and diameter on stress distribution: a finite element analysis. J. Prosthet. Dent.

[46] Kheiralla L.S., Younis J.F. (2014). Peri-implant biomechanical responses to standard, short-wide, and mini implants supporting single crowns under axial and off-axial loading (an in vitro study). J. Oral Implantol..

[47] Kong L., Sun Y., Hu K. (2008). Bivariate evaluation of cylinder implant diameter and length: a three-dimensional finite element analysis. J. Prosthodont..

[48] Kong L., Gu Z., Li T. (2009). Biomechanical optimization of implant diameter and length for immediate loading: a nonlinear finite element analysis. Int. J. Prosthodont. (IJP).

[49] Kong L., Gu Z., Hu K. (2009). Optimization of the implant diameter and length in type B/2 bone for improved biomechanical properties: a three-dimensional finite element analysis. Adv Eng Softw.

[50] Li T., Kong L., Wang Y. (2009). Selection of optimal dental implant diameter and length in type IV bone: a three-dimensional finite element analysis. Int. J. Oral Maxillofac. Surg..

[51] Li T., Hu K., Cheng L. (2011). Optimum selection of the dental implant diameter and length in the posterior mandible with poor bone quality-A 3D finite element analysis. Appl. Math. Model..

[52] Moriwaki H., Yamaguchi S., Nakano T., Yamanishi Y., Imazato S., Yatani H. (2016). Influence of implant length and diameter, bicortical anchorage, and sinus augmentation on bone stress distribution: three-dimensional finite element analysis. Int. J. Oral Maxillofac. Implants.

[53] Niroomand M.R., Arabbeiki M. (2019). Statistical analysis of implant and thread parameters effects on dental implant stability and bone resorption using central composite design method. Proc. Inst. Mech. Eng. H.

[54] Niroomand M.R., Arabbeiki M. (2020). Effect of the dimensions of implant body and thread on bone resorption and stability in trapezoidal threaded dental implants: a sensitivity analysis and optimization. Comput. Methods Biomech. Biomed. Eng..

[55] Özil E., Özkan N., Keskin M. (2023). The effect of short implants placed in the posterior region on tilted implants in the 'All-On-Four' treatment concept: a three-dimensional finite element stress analysis. Comput. Methods Biomech. Biomed. Eng..

[56] Park J., Park S., Kang I. (2022). Biomechanical effects of bone quality and design features in dental implants in long-term bone stability. J Comput Des Eng.

[57] Pellizzer E.P., Verri F.R., de Moraes S.L., Falcón-Antenucci R.M., de Carvalho P.S., Noritomi P.Y. (2013). Influence of the implant diameter with different sizes of hexagon: analysis by 3-dimensional finite element method. J. Oral Implantol..

[58] Petrie C.S., Williams J.L. (2005). Comparative evaluation of implant designs: influence of diameter, length, and taper on strains in the alveolar crest. A three-dimensional finite-element analysis. Clin. Oral Implants Res..

[59] Robau-Porrua A., Pérez-Rodríguez Y., Soris-Rodríguez L.M., Pérez-Acosta O., González Z.E. (2020). The effect of diameter, length and elastic modulus of a dental implant on stress and strain levels in peri-implant bone: a 3D finite element analysis. Bio Med. Mater. Eng..

[60] Raaj G., Manimaran P., Kumar C.D., Sadan D.S., Abirami M. (2019). Comparative evaluation of implant designs: influence of diameter, length, and taper on stress and strain in the mandibular segment-A three-dimensional finite element analysis. J. Pharm. BioAllied Sci..

[61] Sheikhan E., Kadkhodazadeh M., Amid R., Lafzi A. (2022). Interactive effects of five dental implant design parameters on the peak strains at the interfacial bone: a finite element study. Int. J. Oral Maxillofac. Implants.

[62] Shinya A., Ishida Y., Miura D., Shinya A. (2021). The effect of implant length and diameter on stress distribution around single implant placement in 3D posterior mandibular FE model directly constructed form in vivo CT. Materials.

[63] Ueda N., Takayama Y., Yokoyama A. (2017). Minimization of dental implant diameter and length according to bone quality determined by finite element analysis and optimized calculation. J Prosthodont Res.

[64] Baggi L., Di Girolamo M., Vairo G., Sannino G. (2013). Comparative evaluation of osseointegrated dental implants based on platform-switching concept: influence of diameter, length, thread shape, and in-bone positioning depth on stress-based performance Comput. Math Methods Med.

[65] Ausiello P., Tribst J.P.M., Ventre M. (2021). The role of cortical zone level and prosthetic platform angle in dental implant mechanical response: a 3D finite element analysis. Dent. Mater..

[66] Anitua E., Larrazabal Saez de Ibarra N., Morales Martín I., Saracho Rotaeche L. (2021). Influence of dental implant diameter and bone quality on the biomechanics of single-crown restoration. A finite element analysis. Dent. J..

[67] Geramy A., Rokn A., Keshtkar A., Monzavi A., Hashemi H.M., Bitaraf T. (2018). Comparison of short and standard implants in the posterior mandible: a 3D analysis using finite element method. J. Dent..

[68] García-Braz S.H., Prados-Privado M., Zanatta L.C.S., Calvo-Guirado J.L., Prados-Frutos J.C., Gehrke S.A. (2019). A finite element analysis to compare stress distribution on extra-short implants with two different internal connections. J. Clin. Med..

[69] Menacho-Mendoza E., Cedamanos-Cuenca R., Díaz-Suyo A. (2022). Stress analysis and factor of safety in three dental implant systems by finite element analysis. Saudi Dent J.

[70] Bordin D., Cury A.A.D.B., Faot F. (2019). Influence of abutment collar height and implant length on stress distribution in single crowns. Braz. Dent. J..

